# Unmasking the Silent Invader: A Rare Case of Oral Mucosal Malignant Melanoma With Rapid Multisystem Dissemination

**DOI:** 10.7759/cureus.107956

**Published:** 2026-04-29

**Authors:** Muhammad A Zahid, Humna Rashid, Saleh Khurshied, Maryam Mukhtar, Hadia Sohail

**Affiliations:** 1 Ophthalmology, Monash Health, Clayton, AUS; 2 Otolaryngology - Head and Neck Surgery, Pakistan Institute of Medical Sciences, Islamabad, PAK

**Keywords:** amelanotic melanoma, case presentation, case reporting, histopathology, oral cancers

## Abstract

Oral mucosal melanoma is a rare malignancy of the oral cavity and a small subset of melanomas overall, characterized by an aggressive clinical course and diagnostic challenges. These challenges are particularly pronounced in amelanotic variants due to the lack of pigmentation and their histological resemblance to other spindle cell and epithelial tumors. This report describes a 47-year-old man who presented with a progressively enlarging ulcero-exophytic lesion involving the maxilla and hard palate over nine months, accompanied by cervical lymphadenopathy and rapid development of brain metastasis. Initial histopathological evaluation suggested a spindle cell malignancy; however, immunohistochemical analysis demonstrated positivity for S-100, SOX10, and Melan-A, confirming malignant melanoma. This diagnosis was further supported by repeat biopsy and histopathological findings. Despite treatment with pembrolizumab, the disease progressed rapidly both locally and distantly, necessitating palliative care. This case highlights the persistent challenges in diagnosing oral mucosal melanoma due to its atypical clinical and pathological features, emphasizes the critical role of immunohistochemistry in achieving an accurate diagnosis, and underscores the generally poor prognosis despite current therapeutic options. It also reinforces the importance of early detection and timely multidisciplinary management.

## Introduction

Mucosal melanoma is one of the rarest malignancies among head and neck cancers and represents a biologically aggressive tumor arising from melanocytes of the mucosal epithelium. It accounts for less than 2% of all melanomas and an even smaller proportion of oral cavity cancers, with a very low incidence in the general population [1]. Unlike cutaneous melanoma, mucosal melanoma is not associated with ultraviolet radiation exposure and demonstrates distinct genetic and clinical behavior. Due to these characteristics, it is often diagnosed at an advanced stage because of delayed recognition [2].

The oral cavity is among the most commonly affected sites within mucosal melanoma, particularly the hard palate and maxillary gingiva. However, due to its rarity and variable clinical presentation, oral mucosal melanoma is frequently misdiagnosed as inflammatory lesions, benign vascular proliferations, or poorly differentiated carcinomas [3]. This diagnostic challenge is further amplified in amelanotic variants, where the absence of pigmentation removes a key clinical clue, often leading to delayed biopsy and staging [3].

Histologically, mucosal melanoma exhibits significant morphological heterogeneity, including epithelioid, spindle cell, and desmoplastic patterns. In amelanotic lesions, immunohistochemistry plays a crucial role in diagnosis, with markers such as S-100, SOX10, HMB-45, and Melan-A serving as key tools for confirming melanocytic origin [4].

Clinically, oral mucosal melanoma is characterized by aggressive local invasion and early metastatic potential. Cervical lymph node involvement is frequently present at diagnosis and is an important adverse prognostic factor [5]. Despite multimodal treatment strategies, including surgery, radiotherapy, and immunotherapy, outcomes remain poor compared to cutaneous melanoma due to its distinct tumor biology and reduced responsiveness to immunotherapy [6,7].

Given its rarity, subtle presentation, and aggressive course, each case of oral mucosal melanoma provides valuable insight into its diagnostic challenges and clinical behavior. This report describes a rare case of gingival amelanotic melanoma with spindle cell morphology, initially misleading histopathological features, and rapid systemic progression despite immunotherapy.

## Case presentation

A 47-year-old man from Punjab, Pakistan, with a 25-year history of cigarette smoking (10 pack-years) and no known comorbidities, presented to the Ear, Nose, and Throat (ENT) outpatient department (OPD) with a nine-month history of a non-healing, progressively enlarging ulcerated lesion. The lesion originated on the palate and gradually extended toward the upper lip. The patient reported mild associated pain, difficulty chewing, loosening of teeth, and intermittent bleeding from the lesion, which had become more severe in recent weeks. He also reported clinically significant, though undocumented, weight loss. There was no history of prior malignancy, radiation exposure, or family history of cancer. No other associated ENT symptoms were reported.

**Figure 1:**
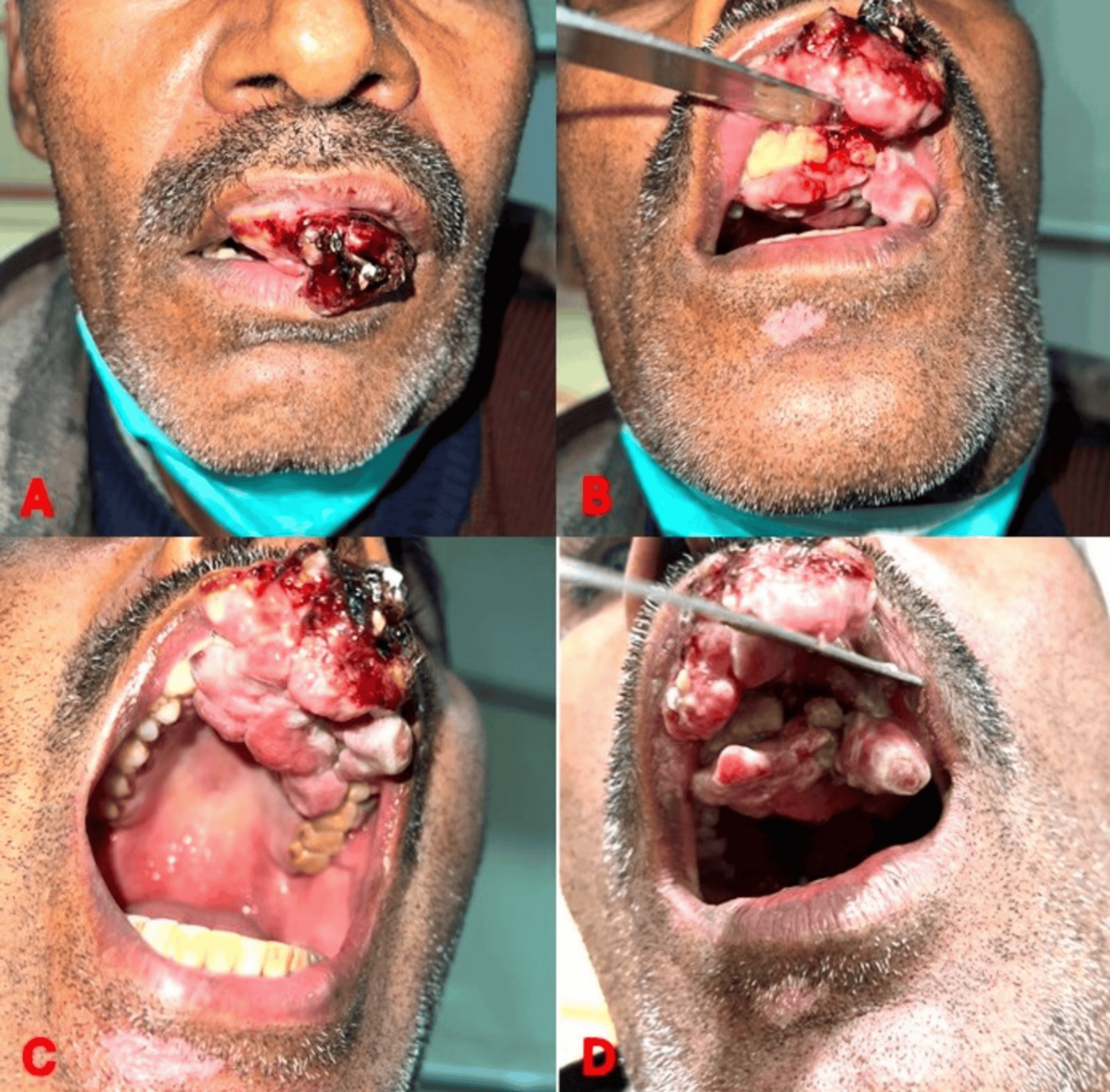
Current clinical presentation of the patient These images show the current clinical presentation at the ninth month of the disease following immunotherapy, demonstrating no response and progressive enlargement of the lesion, as seen from different angles (A-D).

On physical examination, a 5 × 6 cm ulcero-exophytic lesion was observed, extending from the left upper first molar to the right lateral incisor, involving the upper gingivobuccal sulcus and adjacent hard palate, while sparing the upper lip. The lesion was irregularly shaped, with everted margins and covered by pinkish-pale mucosa, with focal areas of subtle pigmentation on close inspection, along with surface ulceration and necrosis. It was firm in consistency, mildly tender, and bled on contact. Oral hygiene was poor, with loosening of teeth. Cranial nerve examination was normal, with no sensory deficits. The patient appeared cachectic and pale. These findings are illustrated in Figures 1a-1d. Neck examination revealed bilateral cervical lymphadenopathy. On the left side, a level IB lymph node measuring 4 × 3 cm was noted, along with smaller nodes at levels IIA (1.5 × 1.5 cm) and III (1 × 1 cm); all were hard, fixed, and non-tender. On the right side, a lymph node measuring 1.5 × 1.5 cm was palpable at level II, with similar characteristics.

Baseline laboratory investigations, including complete blood count, renal function tests, and liver function tests, were within normal limits. Serum lactate dehydrogenase and erythrocyte sedimentation rate (ESR) were mildly elevated. Viral screening for hepatitis B, hepatitis C, and human immunodeficiency virus was negative. A chest radiograph showed no pulmonary lesions, and baseline cardiac evaluation, including electrocardiography, was unremarkable. These findings are summarized in Table 1.

**Table 1:** Summary of the patient’s blood investigations

Lab investigation	Patient value	Normal range
Hemoglobin	13.1 g/dL	13-17 g/dL
Total leukocyte count	8.28 x 10^9^/L	4-10 x 10^9^/L
Erythrocyte sedimentation rate (ESR)	34 mm	<20 mm (Westergren method)
Bilirubin total	18.5 µmol/L	5.1-20.5 µmol/L
Alanine aminotransferase-glutamic-pyruvic transaminase (ALT-SGPT)	37.4 U/L	4-42 U/L
Alkaline phosphatase	53.6 U/L	40-130 U/L
Urea (S)	4.3 mmol/L	2.2-7.1 mmol/L
Creatine	76.3 µmol/L	53-114.9 µmol/L
Sodium	138.7 mmol/L	136-146 mmol/L
Potassium (S)	4.3 mmol/L	3.5-5.1 mmol/L
Lactate dehydrogenase (LDH)	336 U/L	100-280 U/L
Hepatitis B surface antigen (HbsAg) (screening method)	Negative	Negative
Hepatitis C virus antibody test (anti-HCV) (screening method)	Negative	Negative

**Figure 2:**
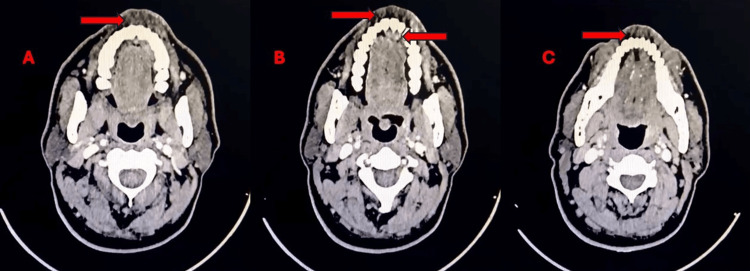
(A-C) Contrast-enhanced computed tomography (CECT) scan of head and neck (axial view) showing enhancing mucosal thickening in the left upper gingival region (red arrows)

Contrast-enhanced computed tomography showed enhancing mucosal thickening in the left upper gingival region, along with sub-centimetric cervical lymphadenopathy and no evidence of distant metastasis, as shown in Figure 2.

The patient’s past history revealed that in March 2025, he underwent an incisional biopsy of the lesion at the dental department, when the lesion was confined to the hard palate. Histopathological examination showed elongated malignant spindle cells arranged in nests and whorls, with hyperchromatic nuclei, suggestive of a malignant spindle cell neoplasm. Immunohistochemical analysis demonstrated strong positivity for S-100 protein, SOX10, and Melan-A, supporting a diagnosis of malignant melanoma. Tumor cells were negative for cytokeratin, excluding poorly differentiated carcinoma. The patient was subsequently referred to the oncology department and received three cycles of immunotherapy with pembrolizumab, with the last cycle administered in October 2025.

**Figure 3:**
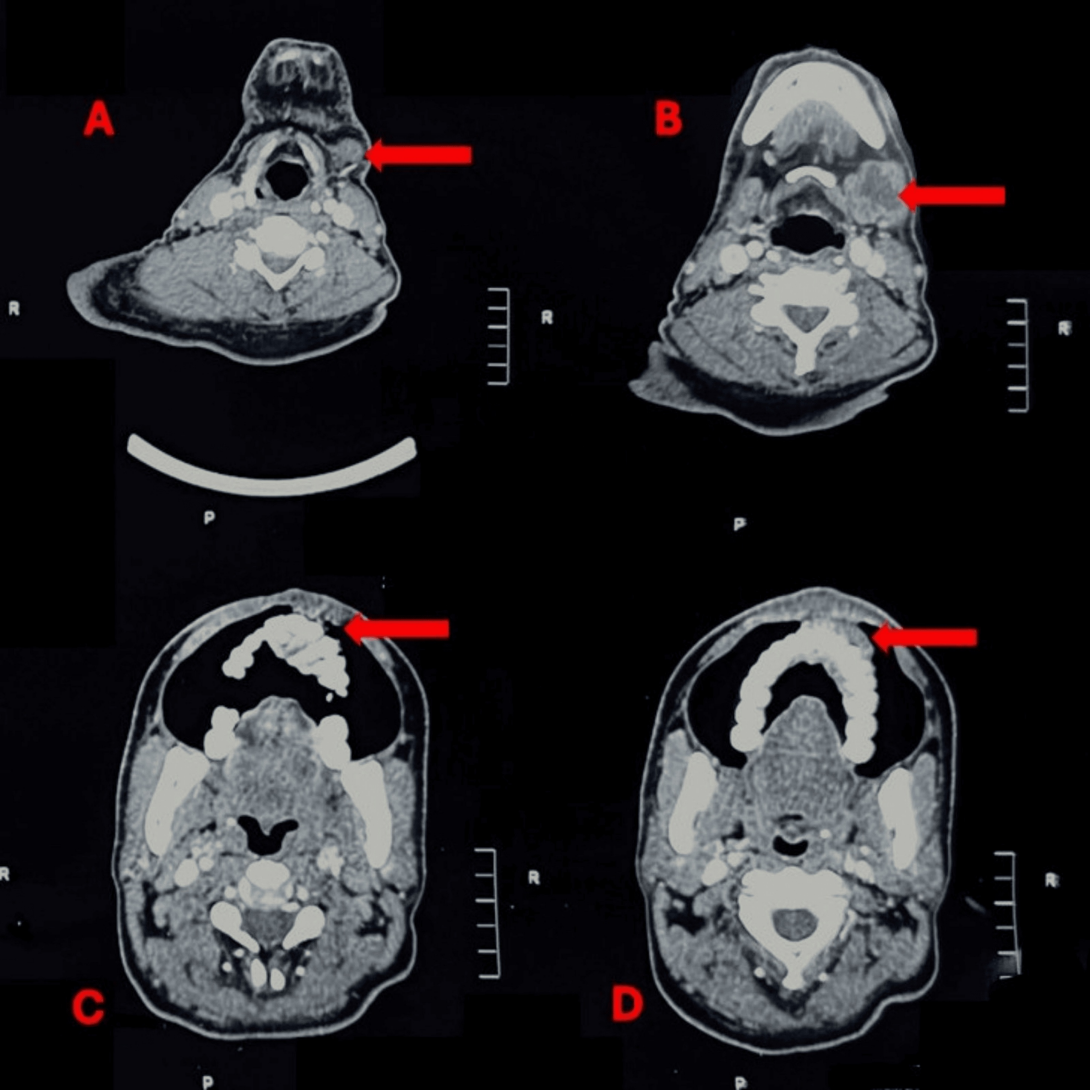
Post-immunotherapy contrast-enhanced computed tomography scan (CECT) of head and neck (axial view) (A, B) Multiple enlarged cervical lymph nodes with loss of fatty hilum and areas of central necrosis (red arrows). (C, D) Infiltrative soft tissue mass involving the hard palate with erosion of the adjacent maxillary bone and extension into the gingivobuccal sulcus (red arrows).

Post-immunotherapy clinical reassessment showed no reduction in tumor size. Follow-up imaging demonstrated disease progression, with increasing local infiltration and persistent nodal involvement. Contrast-enhanced computed tomography of the head and neck revealed an infiltrative soft tissue mass involving the hard palate, with erosion of the adjacent maxillary bone and extension into the gingivobuccal sulcus. Multiple enlarged cervical lymph nodes with loss of fatty hilum and areas of central necrosis were noted, consistent with metastatic involvement. No distant metastases were identified on initial staging. These findings were suggestive of primary resistance to immunotherapy, as shown in Figure 3.

**Figure 4:**
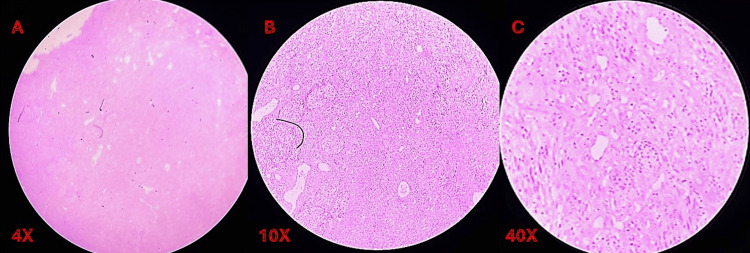
Hematoxylin and eosin (H&E)-stained photomicrographs at different magnifications Histopathology revealed pigmented, pleomorphic, epithelioid, and spindle cells in a nesting to sheet-like pattern and desmoplastic stroma, characteristic of mucosal malignant melanoma (A-C).

Given the atypical clinical appearance, an incisional biopsy was repeated in November 2025. Histopathological examination revealed pigmented, pleomorphic epithelioid and spindle cells arranged in nests and sheet-like patterns within a desmoplastic stroma, confirming the diagnosis of mucosal malignant melanoma (Figure 4).

**Figure 5:**
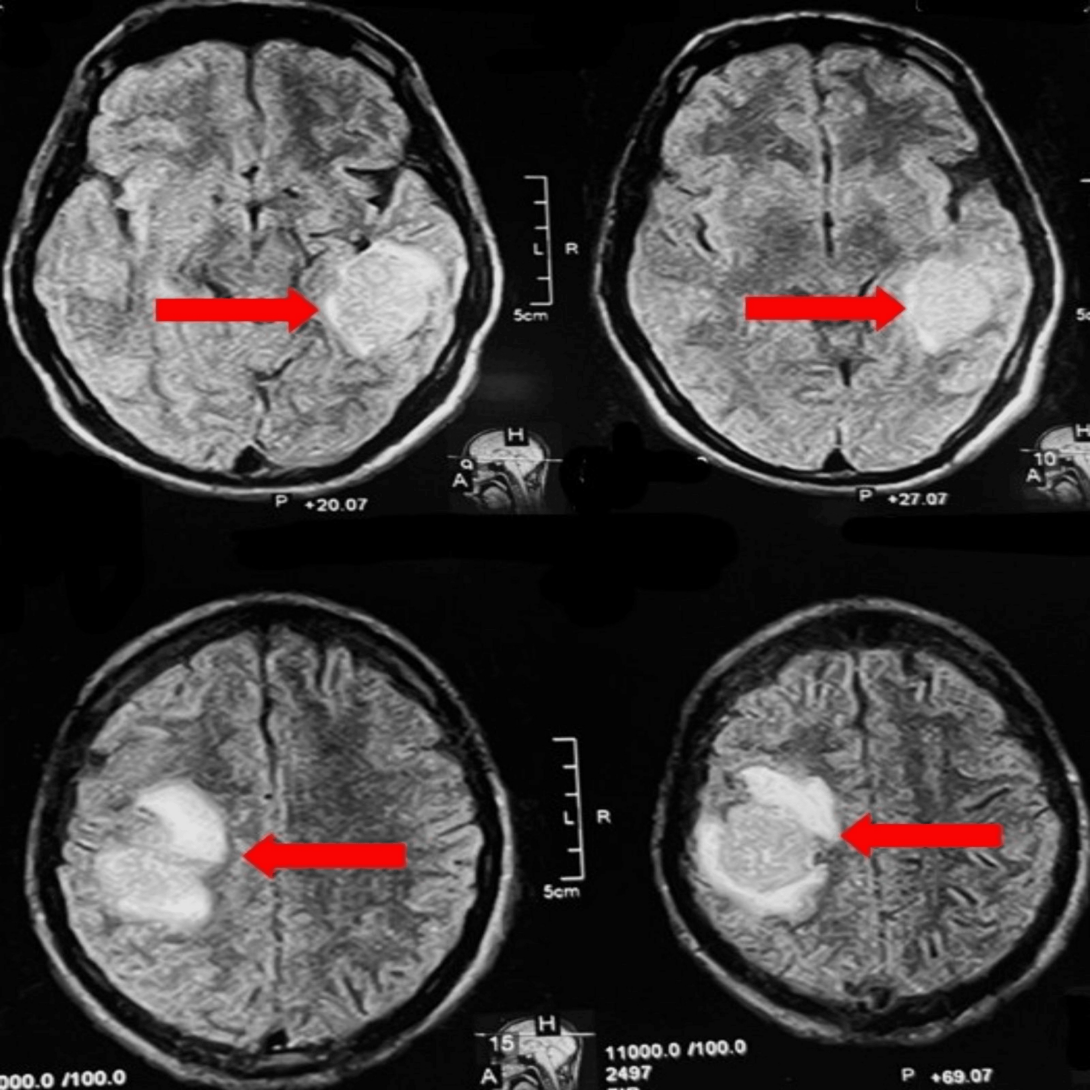
Contrast-enhanced magnetic resonance imaging of the brain (axial view) showing bilateral space-occupying lesions indicating metastases (red arrows)

The patient was subsequently planned for maxillectomy with bilateral neck dissection. However, during preoperative hospitalization, he developed progressive left upper limb weakness over a period of 10 days. Neurological examination revealed reduced motor power (3/5) in the left upper limb, with preserved sensory function. Following neurosurgical consultation, contrast-enhanced magnetic resonance imaging of the brain revealed two heterogeneous enhancing intra-axial lesions with surrounding vasogenic edema, consistent with metastatic disease (Figure 5).

In view of distant metastasis, disease progression despite immunotherapy, and the overall advanced stage, the case was discussed at a multidisciplinary tumor board meeting. The disease was deemed unresectable, and a decision was made to proceed with external beam radiotherapy and palliative care.

## Discussion

The amelanotic variant of mucosal malignant melanoma of the oral cavity is a rare and diagnostically challenging subset of melanomas, accounting for less than 1% of all melanomas and a small fraction of oral malignancies [1,2]. The absence of melanin pigmentation frequently leads to diagnostic confusion, as these lesions may clinically resemble benign inflammatory conditions, pyogenic granulomas, poorly differentiated squamous cell carcinoma, or other spindle cell neoplasms [2,3]. This often results in delayed diagnosis, with patients typically presenting at an advanced stage with local invasion or metastasis, contributing to a poor prognosis.

The clinical features observed in our case are consistent with previously reported patterns. The patient, a 47-year-old man, presented with a non-pigmented exophytic lesion involving the maxillary gingiva and hard palate, associated with tooth mobility and cervical lymphadenopathy. The literature indicates that oral mucosal melanoma most commonly affects individuals in the fifth to seventh decades of life, with a slight male predominance [1,3]. The hard palate and maxillary gingiva are the most frequently involved sites, likely due to a higher concentration of melanocytes in these regions [2]. Similar to our case, lesions are often described as erythematous or pink masses, with bleeding and tooth mobility reflecting advanced disease [3,7]. The relatively younger age at presentation and extensive local disease in our patient further highlight the aggressive nature and often delayed detection of this entity, which rendered the disease untreatable in this case.

Histopathological diagnosis of amelanotic melanoma remains challenging due to its wide morphological variability. The spindle cell pattern, as seen in our case, is one of the most frequently reported variants and can closely mimic sarcomas or other spindle cell malignancies [3]. In our patient, the initial biopsy suggested a malignant spindle cell tumor, illustrating this diagnostic pitfall. Similar challenges have been reported in prior case studies, where immunohistochemistry was essential for definitive diagnosis [6,8]. Immunohistochemical markers such as S-100, SOX10, HMB-45, and Melan-A are highly sensitive for melanocytic differentiation and are crucial in distinguishing melanoma from other malignancies [4]. In our case, immunohistochemistry showing strong positivity for S-100, SOX10, and Melan-A, along with cytokeratin negativity, confirmed melanocytic origin and excluded epithelial malignancy.

Oral mucosal melanomas are biologically aggressive tumors characterized by early metastatic spread. The presence of cervical lymph node metastasis at initial presentation, as in our case, is reported in a substantial proportion of patients and is strongly associated with poor prognosis and reduced overall survival [2,5]. Despite initial staging showing no distant metastasis, our patient developed brain metastases within a short period, reflecting the known aggressive behavior and early dissemination pattern of mucosal melanoma [1,2].

Management of oral mucosal melanoma remains challenging due to its rarity and the lack of standardized treatment protocols. Surgical excision with clear margins remains the mainstay of treatment for localized disease; however, many patients present at advanced stages where complete resection is not feasible [2,4]. In such cases, multimodal therapy, including radiotherapy and systemic treatment, is employed. Although immunotherapy agents such as pembrolizumab have demonstrated significant benefit in cutaneous melanoma, their efficacy in mucosal melanoma is comparatively limited [7,9]. The lack of response to pembrolizumab in our patient, followed by rapid disease progression and the development of brain metastases, is consistent with previously reported lower response rates in mucosal melanoma and may reflect its distinct tumor biology and lower immunogenicity [7,8].

In a case report by Aziz et al. [10], a similar presentation was described, highlighting the diagnostic difficulty of oral malignant melanoma due to the absence of pigmentation and emphasizing the essential role of immunohistochemistry in establishing the correct diagnosis. Given the aggressive and potentially lethal nature of malignant melanoma, it should be considered in the differential diagnosis of oral lesions, even though its oral occurrence, particularly in the amelanotic form, is rare.

This case highlights a rare presentation of gingival amelanotic melanoma with spindle cell morphology from Pakistan, contributing to the limited regional data on this entity. The absence of pigmentation, histopathological overlap with spindle cell tumors, and atypical clinical presentation resulted in diagnostic delay. This underscores the importance of maintaining a high index of suspicion for amelanotic melanoma in any persistent oral lesion, regardless of pigmentation. Early biopsy, comprehensive immunohistochemical analysis, and a multidisciplinary approach are essential for timely diagnosis; however, prognosis remains poor in advanced disease.

## Conclusions

Oral mucosal melanoma is a rare and aggressive malignancy that poses significant diagnostic and therapeutic challenges. The absence of pigmentation and its variable histopathological appearance often lead to misdiagnosis, particularly when it mimics spindle cell tumors or other oral malignancies. As demonstrated in this case, delayed recognition can result in advanced local disease with early regional and distant metastasis, including involvement of the brain.

This case highlights the importance of maintaining a high index of suspicion for melanoma in any persistent, non-healing oral lesion, regardless of pigmentation. Early biopsy and comprehensive immunohistochemical analysis are essential for accurate diagnosis. Despite multimodal treatment approaches, including surgery, radiotherapy, and immunotherapy, the prognosis remains poor due to its aggressive biological behavior and limited treatment response. Increased clinical awareness and early multidisciplinary intervention are crucial to improving outcomes in this rare but lethal disease.
